# Multifunctional Surface Modification of PDMS for Antibacterial Contact Killing and Drug-Delivery of Polar, Nonpolar, and Amphiphilic Drugs

**DOI:** 10.1021/acsabm.2c00705

**Published:** 2022-11-02

**Authors:** Annija Stepulane, Anand Kumar Rajasekharan, Martin Andersson

**Affiliations:** † Department of Chemistry and Chemical Engineering, Chalmers University of Technology, GothenburgSE-412 96, Sweden; ‡ Amferia AB, Astra Zeneca BioVentureHub c/o Astra Zeneca, Pepparedsleden 1, MölndalSE-431 83, Sweden

**Keywords:** polydimethylsiloxane, antibacterial coating, antimicrobial peptides, hydrogel microparticles, drug delivery

## Abstract

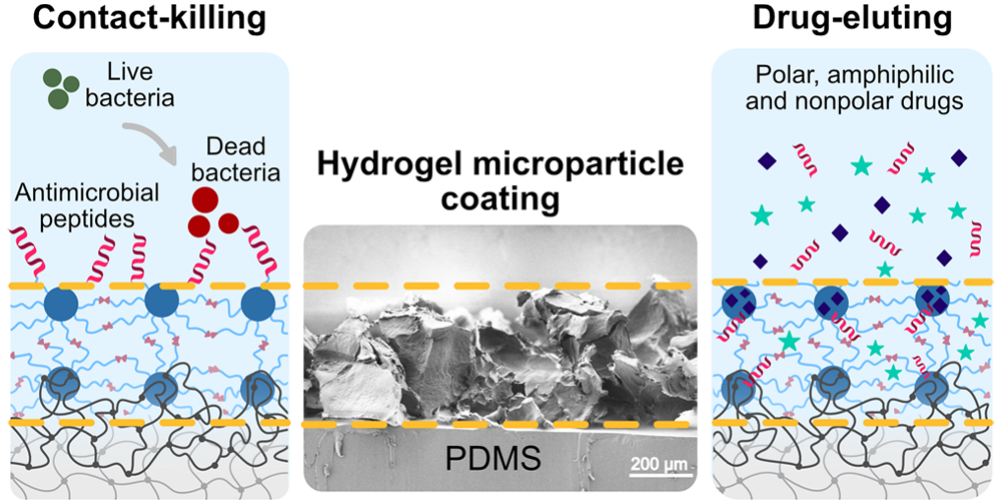

Medical device-associated infections pose major clinical challenges that emphasize the need for improved anti-infective biomaterials. Polydimethylsiloxane (PDMS), a frequently used elastomeric biomaterial in medical devices, is inherently prone to bacterial attachment and associated infection formation. Here, PDMS surface modification strategy is presented consisting of a cross-linked lyotropic liquid crystal hydrogel microparticle coating with antibacterial functionality. The microparticle coating composed of cross-linked triblock copolymers (diacrylated Pluronic F127) was deposited on PDMS by physical immobilization via interpenetrating polymer network formation. The formed coating served as a substrate for covalent immobilization of a potent antimicrobial peptide (AMP), RRPRPRPRPWWWW-NH_2_, yielding high contact-killing antibacterial effect against *Staphylococcus epidermidis* and *Staphylococcus aureus.* Additionally, the coating was assessed for its ability to selectively host polar, amphiphilic, and nonpolar drugs, resulting in sustained release profiles. The results of this study put forward a versatile PDMS modification strategy for both contact-killing antibacterial surface properties and drug-delivery capabilities, offering a solution for medical device-associated infection prevention.

## 1 Introduction

An aging population in combination with advancements in healthcare quality and accessibility worldwide has led to a steady increase in demand for medical devices, with the market expected to grow from $425 billion in 2018 to $615 billion in 2025.^1^ Despite the pervasiveness of various short-term and long-term medical devices, medical device-associated infections, which result from surface colonization of infectious bacteria, have remained a challenge to address. Depending on the function of a device, anatomical site, and the level of invasiveness, infections vary strongly in incidence and severity.^2^ With infection rates ranging from 1% in hip and knee prosthesis to 70–80% in urinary catheters, acquiring an infection leads to an extended or repeated hospitalization along with increased patient morbidity and mortality.^3,4^

The current strategies for addressing medical device-associated bacterial infections often entail the use of prophylactic or therapeutic antibiotics. However, the emergence of antibiotic-resistant bacterial infections and the limited efficiency of systemic antimicrobials against bacterial biofilms^5,6^ hinder efforts to develop antimicrobial biomaterial solutions. As a consequence, the need for antibiotic-free infection prevention strategies has led to extensive research efforts directed toward new and improved antibacterial biomaterial development.

Polydimethylsiloxane (PDMS) is a silicone-based elastomer commonly utilized in biomedical applications due to its excellent properties such as mechanical and chemical stability, tunable elasticity, ease of processability, and good biological compatibility. These properties have facilitated its usage in manufacturing of urinary catheters, medical tubing, shunts, and contact lenses along with numerous types of aesthetic and orthopedic implants.^7,8^ Despite the widespread applicability, pristine PDMS surfaces are still susceptible to irreversible bacterial attachment and biofilm formation, which remain major challenges in PDMS-based medical device performance.^9,10^

To date, a plethora of physicochemical surface modification methods of PDMS have been developed to counteract biomaterial-associated infections via antifouling surfaces, e.g., inhibition of bacterial attachment, or eradication of biofilms by antibacterial or antibiofilm surface coatings.^11^

Antifouling PDMS surfaces that inhibit bacterial attachment can be produced via surface topography alterations, limiting the area available for bacterial attachment by, for example, patterning the surface with micro- and nanofeatures.^12,13^ Additionally, PDMS surface functionalization with moieties known for their antifouling properties like zwitterionic polymer brushes or polyzwitterionic coatings^14,15^ has been reported to restrict bacterial adhesion. An alternative approach entails the impregnation of biocidal agents into the PDMS structure, or biocide immobilization onto the PDMS surface via physisorption or covalent attachment, whereupon bacterial killing is achieved either by contact-killing or biocide eluting properties. Such strategies have shown to give high antibacterial activity against common Gram-positive and Gram-negative bacteria and have been reported by utilizing conventional antibiotics,^16,17^ silver compounds,^18^ antimicrobial peptides (AMP),^19,20^ quaternary ammonium compounds,^21^ and chitosan derivatives^22,23^ to name a few.

In this work, we present a PDMS surface modification strategy by utilizing AMP-functionalized cross-linked lyotropic liquid crystal hydrogel microparticle coating onto PDMS to impart contact-killing antibacterial surface properties alongside localized drug delivery capacity. Here a potent cationic AMP RRPRPRPRPWWWW-NH_2_ (RRP9W4N) has been covalently attached onto cross-linked diacrylate Pluronic F127 (DA-F127) hydrogel microparticles via carbodiimide coupling chemistry followed by subsequent particle immobilization onto PDMS surface.^24^ Cross-linked DA-F127 bulk hydrogels have previously been reported to serve as suitable immobilization platforms for AMP covalent attachment in wound-care applications by exerting strong antibacterial effect against *Staphylococcus epidermidis*, *Staphylococcus aureus*, *Pseudomonas aeruginosa*, methicillin-resistant *S. aureus* (MRSA), and multidrug-resistant *Escherichia coli*.^25^ However, DA-F127 bulk hydrogels lack mechanical integrity, and their use is limited in structurally demanding applications like the PDMS elastomers. In consequence, DA-F127 hydrogel coating is deemed to be a promising surface modification strategy combining the DA-F127 functionality with the PDMS substrate mechanical stability.

Antibacterial hydrogel microparticle formulations utilized in this study were previously developed by E. Blomstrand et al. through top-down methods from bulk cross-linked versions of ordered triblock DA-F127 copolymer.^26^ The amphiphilicity of Pluronic F127 backbone facilitates the polymer self-assembly into ordered lyotropic liquid crystals according to the ternary phase diagram.^27^ Here the cross-linked version of micellar cubic phase was utilized. The particles were embedded onto PDMS substrates via an intermediated thin adhesive film of PDMS prepolymer layer. The amphiphilicity of the lyotropic liquid crystal hydrogel particles facilitated the formation of an interpenetrating polymer network (IPN) between the PDMS and the hydrogel interface. By subsequent curing of the PDMS thin films, a stable particle coating was established. The hydrophobic domains of the micellar cubic structure assisted the physical entrapment in PDMS matrix and prevented delamination, while the hydrophilic domains remained accessible for covalent AMP attachment via carbodiimide coupling chemistry.

To demonstrate complementary functions of the produced microparticle coating on PDMS substrates, the manufactured constructs were investigated for their ability to selectively entrap and release therapeutic agents of different polarity *in vitro*. Due to the Pluronic F127 network amphiphilicity and thermoreversible gelling properties, Pluronic F127 gels have been widely exploited as drug carriers of hydrophilic and hydrophobic therapeutic drugs.^28,29^ In the present study, the DA-F127 microparticle coating was loaded with polar, amphiphilic, and nonpolar model drugs (i.e., vancomycin (VCM) antibiotic, AMP, and ibuprofen (IBP) anti-inflammatory drug) and their release behavior monitored in a proof-of-concept study. To characterize the drug release kinetics and elucidate the release mechanisms, release data were mathematically fitted to zero-order, first-order, Korsmeyer-Peppas, and Higuchi mathematical models.

The present work demonstrates the development of a new type of PDMS surface modification via fabrication of cross-linked DA-F127 lyotropic liquid crystal microparticle coating. The coating was investigated for its physiochemical properties and mechanical stability via contact angle analysis, Raman spectroscopy, X-ray photoelectron spectroscopy, and peel-off tests, revealing a successful PDMS surface modification with durable hydrogel particle coating of hydrophilic properties. Additionally, the particle coating served as a platform for covalent AMP attachment exerting high contact-killing antibacterial effect against *S. epidermidis* and *S. aureus*. A proof-of-concept drug delivery study illustrated the capacity of the particle coating for encapsulation and sustained release of polar (VCM), amphiphilic (AMP), and nonpolar (IBP) drugs. The current findings demonstrate a new method for PDMS surface modification to yield antibacterial surface properties with a complementary drug delivery function.

## 2 Materials and Methods

Unless stated otherwise, all reagents used in this work were provided by Sigma-Aldrich Sweden AB (Stockholm, Sweden) and used as received without further purification.

### 2.1 Hydrogel Particle Preparation

Hydrogel particles were prepared via a top-down approach from bulk DA-F127 hydrogels using a method stated elsewhere.^26^ In brief, bulk hydrogel was prepared by manually mixing 30% w/w of the DA-F127 copolymer powder (DA-PEO_100_-PPO_70_-PEO_100_-DA, provided by Amferia AB) with Milli-Q (MQ) water to form a homogeneous gel. The surfactant–water concentration was chosen due to its ability to form a lyotropic liquid crystalline phase of micellar cubic structure, similar to the nonmodified Pluronic F127.^27^ To facilitate the cross-linking, 0.05% w/w photoinitiator Irgacure 2959 was added to the lyotropic liquid crystal gel with respect to the copolymer weight. The gel was then stored at 4 °C until fully liquified, cast onto a glass mold, and equilibrated in room temperature for 2 h to fully set into micellar cubic phase. Finally, the gel was cross-linked into a solid sheet in a UV chamber (UVP Cross-linker CL-3000, Analytik Jena, Germany) at λ = 302 nm. The sheet was cut into smaller strips and washed in MQ water for 48 h with water change at least twice to wash away any unreacted polymer.

The swollen hydrogel strips were ground into particles using a food processor (MQ 7000X, Braun GmbH, Germany), and the particle size was further reduced by homogenizing the particle suspension with a disperser (T 18 ULTRA-TURRAX, IKA Werke GmbH and Co., Germany). The resulting suspension was suction filtered through cellulose filter (Whatman grade 3, Cytiva Europe GmbH, Sweden) of 6 μm pore size. The particle paste was divided with one part intended as control particles and one part intended for further AMP functionalization to form the antibacterial particles (see Section 2.2). Control particles were placed in a glass beaker, snap frozen in liquid N_2_, and subsequently freeze-dried for 24 h to remove any residual water. The freeze-dried particle powder was manually ground by a mortar and pestle to disperse any particle aggregates that resulted from the drying process and stored in air atmosphere until further use.

### 2.2 AMP Functionalization of Particles

Suction-filtered particles were functionalized with AMP via free carboxyl group activation. One gram of as-prepared particles was dispersed in 5 mL of activation solution consisting of 2 mg/mL of 1-ethyl-3-(3-(dimethylamino)propyl) carbodiimide (EDC) and 2 mg/mL *N*-hydroxysuccinimide (NHS) solutions in 0.5 M 2-(*N*-morpholino) ethanesulfonic acid buffer (MES, pH 6) and maintained under constant stirring for 30 min. Afterward, the activated particles were repeatedly suction filtered and washed with a copious amount of MQ water to remove any excess activation reagents. The activated particle paste was dispersed in 5 mL of 400 μM RRP9W4N AMP powder (Amferia AB, Sweden) dissolved in phosphate buffered saline (PBS, pH 7.4) and continuously stirred for 2 h to facilitate covalent AMP attachment via peptide bond formation. After 2 h, AMP-functionalized particle suspension was suction filtered, and the filtrate was sampled for further analysis to quantify AMP uptake by the particles. Ultimately, AMP-functionalized particles were washed through the filter with a copious amount of MQ water to remove any free peptide, filtered, frozen in liquid N_2_, freeze-dried, and stored until further use.

The AMP uptake in particles was quantified using a UV–vis spectrophotometer (Multiskan GO, Thermo Fisher Scientific, MA, USA) at λ = 280 nm by calculating the difference in peptide concentration in the filtrate and the AMP solution using a standard curve.

### 2.3 Hydrogel Particle Coating on PDMS

PDMS substrates were prepared from Sylgard 184 (Dow Corning, MI, USA) silicone elastomer kit by mixing it according to the manufacturer’s instructions. Ten parts base agent were thoroughly mixed with one part curing agent to form a prepolymer gel. The prepolymer gel was poured into a glass Petri dish (10 mm Ø, 5 mm depth) and degassed in a vacuum desiccator to remove any air bubbles. The mixture was cured in an oven at 120 °C for 45 min until fully cross-linked. The resulting PDMS sheets were cut into 18 × 18 mm^2^ sized squares with a microtome blade, washed in 95% ethanol, and dried under a stream of N_2_ gas.

Subsequently, a new batch of Sylgard 184 prepolymer was mixed according to the instructions and degassed in a vacuum desiccator to remove air bubbles for approximately 10 min. Immediately after the degassing, prepolymer was deposited onto the PDMS substrates (50 ± 5 mg per substrate), placed on a spin coater platform (SPIN150-v3, SPS-Europe B.V., The Netherlands), and rotated at a constant spinning speed for 3 min with an acceleration rate of 500 rpm/s. Samples were prepared at different spinning speeds varied between 1000 to 6000 rpm in increments of 1000 rpm. Thereafter, the resulting PDMS surface was fully covered with the freeze-dried hydrogel particle powder until the underlying PDMS substrate was not visible to the naked eye, allowing for PDMS prepolymer absorption into the hydrophobic domains of the particle structure. Although 100% surface coverage could not be achieved by the deposition of the freeze-dried particles, following swelling in aqueous media, near total surface coverage was achieved visible to the naked eye and with the light microscope.

Two groups of samples were prepared with either control particles or AMP-functionalized particles. Finally, the PDMS substrates with the particle coating were cured in an oven at 37 °C for 24 h, allowing for PDMS film cross-linking and particle entrapment in the PDMS matrix by formation of an interpenetrating polymer network between the PDMS and DA-F127. The resulting materials were flushed with a stream of N_2_ gas to remove any loosely attached particles and stored in air atmosphere until further use.

The manufactured materials of hydrogel particle coatings on PDMS substrates are hereafter referred to as the “coatings”, with AMP-modified particle coating designated as “AMP particle coating” and AMP-free hydrogel particle coating designated as “control particle coating”.

### 2.4 Characterization of Coating

Unless stated otherwise, all coating characterization experiments were conducted on as-prepared freeze-dried particle coatings.

#### 2.4.1 Coating Morphology and Stability Evaluation

Coating morphology and stability were investigated to assess particle adhesion and detachment. Coating morphology and entrapment in the PDMS matrix were examined using a stereo microscope (Stemi 508, Carl Zeiss AG, Germany) in as-prepared state, as well as rehydrated state using MQ water or safranine dye for increased contrast.

Particle size distribution of the as-prepared coatings was measured from stereomicroscope images. Three-hundred randomly selected particles were measured from coatings prepared at different spin speeds. The particle size was measured as equivalent to particle projection diameter and the number-based size distribution calculated.

Scotch tape (Magic Tape, 3M, MI, USA) peel-off method was used to qualitatively assess the as-prepared coating stability in dry state. The tape was manually applied to the dry coating surface and peeled-off in a 90° angle removing any loosely bound particles. The procedure was repeated four times until no additional particle detachment was observed.

#### 2.4.2 Water Contact Angle (WCA)

The wetting properties of the particle coating in as-prepared state were analyzed with optical tensiometer (Theta, Attension, Finland) and compared to pristine PDMS. The tensiometer was operated in static contact angle mode with manual Hamilton syringe dispenser using MQ water. A high-resolution camera was employed to record water droplet spreading on the sample surfaces, and OneAttension software was utilized for image analysis and contact angle determination. All measurements were averaged over three samples per type.

#### 2.4.3 Raman Spectroscopy

Chemical characterization was performed on the as-prepared particle coatings using confocal Raman microscope (Alpha300 R, WITec, Germany) with a laser excitation wavelength of 532 nm at 10 mW power. Samples were analyzed under a 50× objective, with 30 accumulations per measurement point. Pure AMP powder was included in the analysis to probe for the characteristic chemical groups in the peptide structure.

#### 2.4.4 X-ray Photoelectron Spectroscopy (XPS)

Elemental composition of the as-prepared coating surface was determined using scanning XPS microprobe (PHI 5000 VersaProbe III, Ulvac-PHI Inc., Japan) with a monochromatic Al Kα X-ray source. Samples were mounted on a sample stage and sample surface scanned over an area of 400 × 500 μm^2^, obtaining survey scans between 0–1100 eV at a step size of 0.4 eV/step and high-resolution N 1s scans in the region of interest at a step size of 0.1 eV/step, respectively. Silver ion flood gun was used to compensate for sample charging.

#### 2.4.5 Scanning Electron Microscopy (SEM)

Morphology and adhesion of the coating were investigated using SEM (Leo Ultra 55, Carl Zeiss AG, Germany) at 2 kV accelerating voltage. Cross-sections of the as-prepared coated substrates were sliced to 1 mm thickness with a microtome blade, samples mounted on sample stages and sputter coated with gold for 1 min at 10 mA for enhanced imaging.

Additionally, SEM was used to study the particle coatings after exposure to bacteria to understand bacteria–particle interaction and observe any change in bacteria morphology. Coating samples were first rehydrated in phosphate buffered saline (PBS) followed by incubation in 10^8^ colony forming units per mililiter (CFU/mL) *S. epidermidis* suspension in 10% v/v TSB overnight. After 18 h, samples were washed three times with 1 mL of PBS. Bacteria were fixed in 4% formaldehyde solution (VWR International AB, Sweden) for 2 h at room temperature. The samples were then subjected to a dehydration procedure in an ethanol solution gradient (20%, 50%, 70%, 85%, and 99.5%) for 15 min per step, followed by an immersion in 50% v/v hexamethyldisilazane (HDMS) solution in ethanol for 30 min and a final step in 100% HDMS. Samples were placed in a fume hood until fully air-dried and sputter coated with gold prior the SEM analysis.

### 2.5 Antibacterial Activity of Coating

Prior to the antibacterial evaluation, the coatings were rehydrated in PBS and cut in 8 mm Ø sized disks with a biopsy punch. Three groups of samples were prepared for microbiology analysis: AMP-free control particle coatings, AMP particle coatings, and pristine PDMS substrates, which served as a negative control. Prior to the analysis, PDMS substrates were cut to size, rinsed in 70% ethanol, and dried under a stream of N_2_ gas.

Antibacterial activity of the AMP particle coatings was investigated against two Gram-positive bacteria strains, i.e., *S. epidermidis* CCUG 39508 and *S. aureus* CCUG 10778. A single colony of bacteria was dispersed in 4 mL of tryptic soy broth (TSB) and incubated static at 37 °C until mid log growth stage was reached, determined by measuring the optical density at 600 nm (OD 0.55–0.70) and estimated to correspond to 10^9^CFU/mL. The bacteria suspension was then diluted to 10^6^ CFU/mL in 10% v/v TSB in PBS solution. The test samples were placed in separate wells of a sterile 24-well plate and 1 mL of 10^6^ CFU/mL bacteria suspension was pipetted on top of each sample. The samples were incubated static at 37 °C for 1 h, following which the bacteria suspension was carefully aspirated and wells replenished with pure 10% v/v TSB. Afterward, the samples were returned to the incubator and incubated static overnight for approximately 18 h.

The next day, bacteria suspension was aspirated, and samples were washed three times with 1 mL of PBS to remove any planktonic bacteria. Subsequently, individual samples were placed in tubes containing 1 mL of PBS and vortexed for 2 min at 3000 rpm to detach surface-adhered bacteria. The vortexed suspension was serially diluted 10-fold and triplicates of 10 μL aliquots of the vortexed suspension, and the serial dilutions were pipetted onto brain–heart infusion agar plates. The plates were incubated at 37 °C overnight and retrieved the following day for colony counting. All tests were repeated three times in a triplicate per sample type (*n* = 9).

### 2.6 *In Vitro* Drug Delivery Studies

The particle coating was investigated for its ability to encapsulate and release polar, amphiphilic, and nonpolar drugs *in vitro*. For this purpose, AMP-free hydrogel particle coatings were prepared on PDMS substrates as described in the Materials and Methods section at a spin speed of 1000 rpm and cut to 8 × 15 mm^2^ size. The samples were washed in a copious amount of MQ water for 24 h to remove any residual non-cross-linked polymer and air-dried at 37 °C for 24 h. The samples were loaded with the respective drugs by soaking the samples in separate Eppendorf tubes containing a relevant solvent corresponding to drug polarity, i.e., 1.5 mL of 1% w/v AMP solution in MQ water, 1.5 mL of 1% w/v VCM solution in MQ water, or 1.5 mL of 7% w/v IBP solution in acetone, alternatively. The soaking procedure was carried out for 24 h to reach equilibrium particle swelling, followed by an intermediate sample rinsing step in pure acetone or MQ and final drying step at 37 °C for 24 h.

The VCM- and AMP-loaded samples were eluted in 8 mL of MQ water, while IBP was eluted in 16 mL of 1% w/v aqueous surfactant solution consisting of sodium dodecyl sulfate (SDS) to facilitate its solubility. SDS was used since it is commonly utilized in pharmaceutical research for drug dissolution tests of poorly soluble drugs like IBP^30−32^ as well as being commonly utilized in lyophilic drug formulations to increase their bioavailability *in vivo*.^30,33^ The elution volume was previously determined to yield maximum drug concentration under the saturation limit (min 1/3 of the maximum solubility) in order to maintain the systems under sink conditions. The samples were then placed on a mechanical shaker plate, and the elution media were sampled for UV–vis analysis every 15 min for the first 4 h, then every 30 min for 8 h, followed by once every day until no additional elution signal could be detected. The sampled media were returned to the system after each measurement. The absorbance was measured in quartz cuvettes using a UV–vis spectrophotometer (Multiskan GO, Thermo Fisher Scientific) with the pure elution medium (i.e., MQ water or 1% w/v SDS, respectively) used as a blank. Depending on the type of drug loaded, absorbance signal was registered at λ = 280 nm, λ = 280 nm, or λ = 272 nm for VCM, AMP, or IBP, respectively, and the eluted drug mass was quantified based on previously constructed standard curves. The drug delivery studies were repeated three times per drug type in a triplicate of samples (*n* = 9). Additionally, one drug-free particle coating sample was included in each elution experiment to account for any polymer degradation during the elution process that my interfere with the UV absorption signal. To account for the drug uptake in the PDMS substrates, a single control experiment for each type of drug was performed using coating-free PDMS substrate (*n* = 3).

To evaluate drug release kinetics of VCM, AMP, and IBP delivery from the coatings, the experimental data were mathematically fitted to the following drug delivery models: zero-order, first-order, Korsmeyer-Peppas, and Higuchi models. The fitting was performed using nonlinear least-squares regression and the quality of fit expressed via correlation coefficient (*R*^2^) and root–mean–square error (RMSE) using DDSolver add-in program, which can be used for modeling of dissolution data based on a built-in model library.^34^ Additionally, the release rate constants were calculated, and the Korsmeyer-Peppas release exponent *n* was determined for elucidation of the release mechanism.

### 2.7 Statistical Analysis

Antibacterial activity of all samples in this study was calculated by averaging three experiments (*n* = 9) and results expressed as CFU/cm^2^. Standard deviation was calculated to express the data distribution around the mean. Two-tailed Student’s *t* tests assuming unequal variance were conducted to obtain *p*-values and determine statistical significance. Graphical asterisk designation was introduced for different levels of significance with *, **, and *** corresponding to *p* ≤ 0.05, *p* ≤ 0.01, and *p* ≤ 0.001, respectively.

## 3 Results and Discussion

### 3.1 Particle Coating Preparation

The hydrogel particle coating platform was developed by utilizing cross-linked lyotropic liquid crystal hydrogel microparticles previously developed by our research group.^26^ The amphiphilic nature of DA-F127 triblock chain facilitates the polymer self-assembly into ordered lyotropic liquid crystals (LLC) of distinct hydrophilic and hydrophobic domains.^27^ The polymer–water concentration utilized in this study corresponds to LLC organization into an ordered micellar cubic phase and has been previously reported to be capable of retaining its ordered structure post cross-linking.^35^ In this work, the amphiphilicity of the cross-linked particle network was explored for hydrogel particle entrapment in PDMS matrix, to form robust coatings, while simultaneously retaining the functional properties of the hydrogels.

The main obstacle in production of monolith LLC hydrogel coating onto PDMS lies in the physical adsorption of the hydrophobic PPO copolymer segments to the hydrophobic PDMS surface. Governed by the hydrophobic interactions, the resulting adsorption leads to conformational changes in the DA-F127 structure and consequent altering of the PDMS surface hydrophobicity by PEO chain reorientation. Although a well-known property utilized in PDMS microfluidic surface modifications,^36^ the copolymer adsorption inhibits successful chemical conjugation and monolith anchoring onto PDMS. In the context of the present study, production of LLC hydrogel coatings from non-cross-linked copolymers has limited adhesion potential and suffers from delamination. Therefore, the proposed strategy focuses on coatings of premade cross-linked versions of the self-assembled DA-F127 networks in microparticle formulation to circumvent the issues associated with monolith hydrogel coating delamination along with added benefit of increased coating surface area.

**Figure 1:**
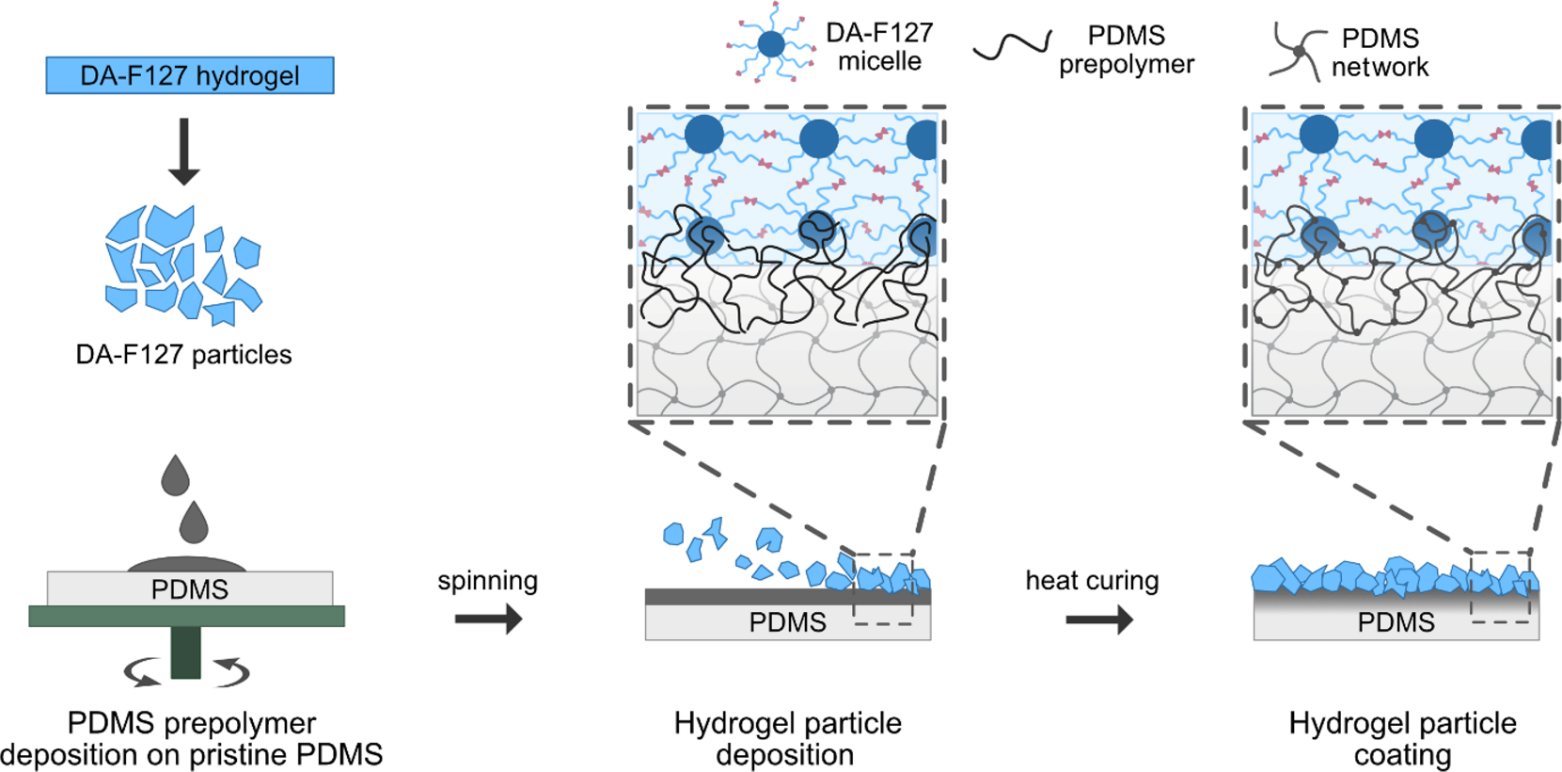
Schematic representation of the proposed hydrogel particle coating onto the PDMS via formation of interpenetrating polymer network. AMP modification step not included.

In the proposed coating strategy (Figure 1), PDMS prepolymer thin film is spin-coated onto PDMS substrate, which was expected to function as the immobilization matrix (or adhesive) between cross-linked and freeze-dried hydrogel particles and the PDMS substrate, establishing a physical IPN network bond between bulk PDMS substrate and the particles. An optimal PDMS film thickness was expected to be sufficient for particle entrapment without delamination, while thin enough to not cover the particles, with loss of functional surface. The hydrophobic nature of the siloxane monomers enabled the penetration of uncured PDMS prepolymer into the hydrophobic domains of the LLC particle structure. By subsequent heat curing, the PDMS thin films were fused with the PDMS substrate, and the particles were physically stabilized onto the PDMS surface via formation of physical network entanglements. The nature of bonding between the PDMS and LLC particles represented a type of sequential interpenetrating polymer network formed between the particles and PDMS interface, with PDMS prepolymer entangling and cross-linking upon hydrosilylation of siloxane monomers in the preformed DA-F127 network matrix.^37,38^ Here the amphiphilicity of the LLC particle network drives the siloxane monomer migration, forming a type of gradient interpenetrating network; however, a more detailed investigation is necessary to elucidate the molecular architecture of the particle–PDMS interface.

### 3.2 Physiochemical Characterization of Particle Coating

**Figure 2:**
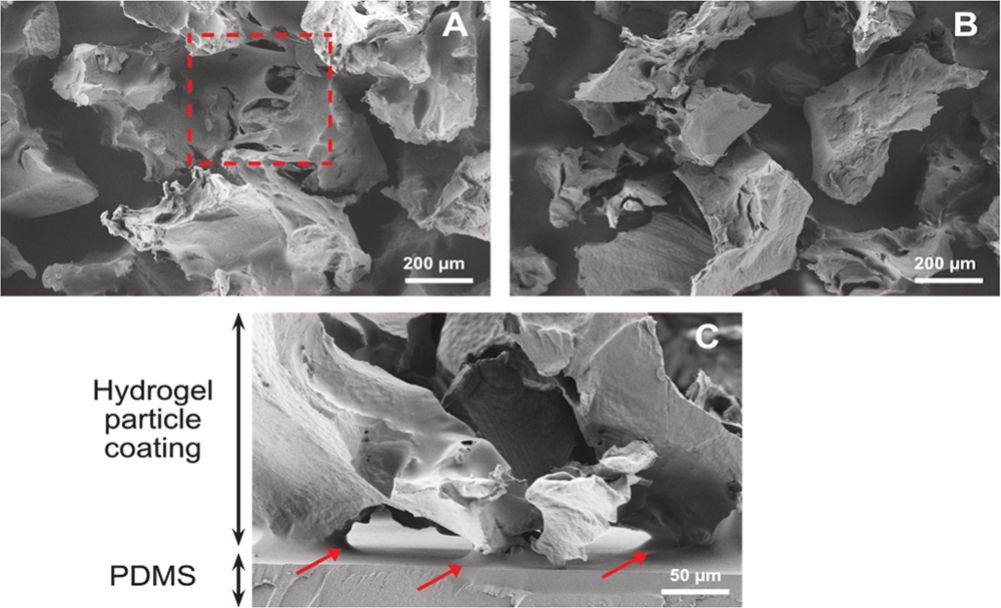
SEM micrographs of the coatings produced at different PDMS film spin coating speeds. (A) Top view of coating produced at 1000 rpm, particle coverage with PDMS at lower spin speeds highlighted in red. (B) Top view of coating produced at 5000 rpm. (C) Cross-section of coating produced at 5000 rpm, visible necking around particles indicated by the red arrows.

An established dependency between the PDMS film thickness and spin rate^39^ was utilized to produce PDMS films of varying thickness, resulting in notable differences among the (a) coated particle load on the final coated material, (b) entrapment depth in the PDMS matrix, and (c) the resulting adhesion strength. Spin rates between 1000 and 6000 rpm were utilized for PDMS film deposition with the produced coatings and cross-sections seen in SEM micrographs in Figure 2. The morphology of the particle coating displayed distinctly asymmetrical particle geometry with jagged profiles, indicative of the particle surface accessibility while also consistent with the top-down manufacturing method of mechanical homogenization.

At the lowest spin rate of 1000 rpm, sections of underlying particles were evidently covered by thick PDMS film as highlighted in red in Figure 2A. Although the coating adhesion may have been stronger at low rpm due to a high polymer network entanglement, there is a notable loss or reduction of available hydrogel surface area. Upon increasing the spin rate to 3000 rpm and above, a majority of the particles coated were clear of any PDMS coverage while still maintaining firm particle attachment and surface availability as exemplified by samples produced at 5000 rpm (Figure 2B). Additionally, PDMS uptake in the hydrogel network structure was maintained as seen from the necking around the particle–PDMS interface in the cross-sectional images (Figure 2C).

**Figure 3:**
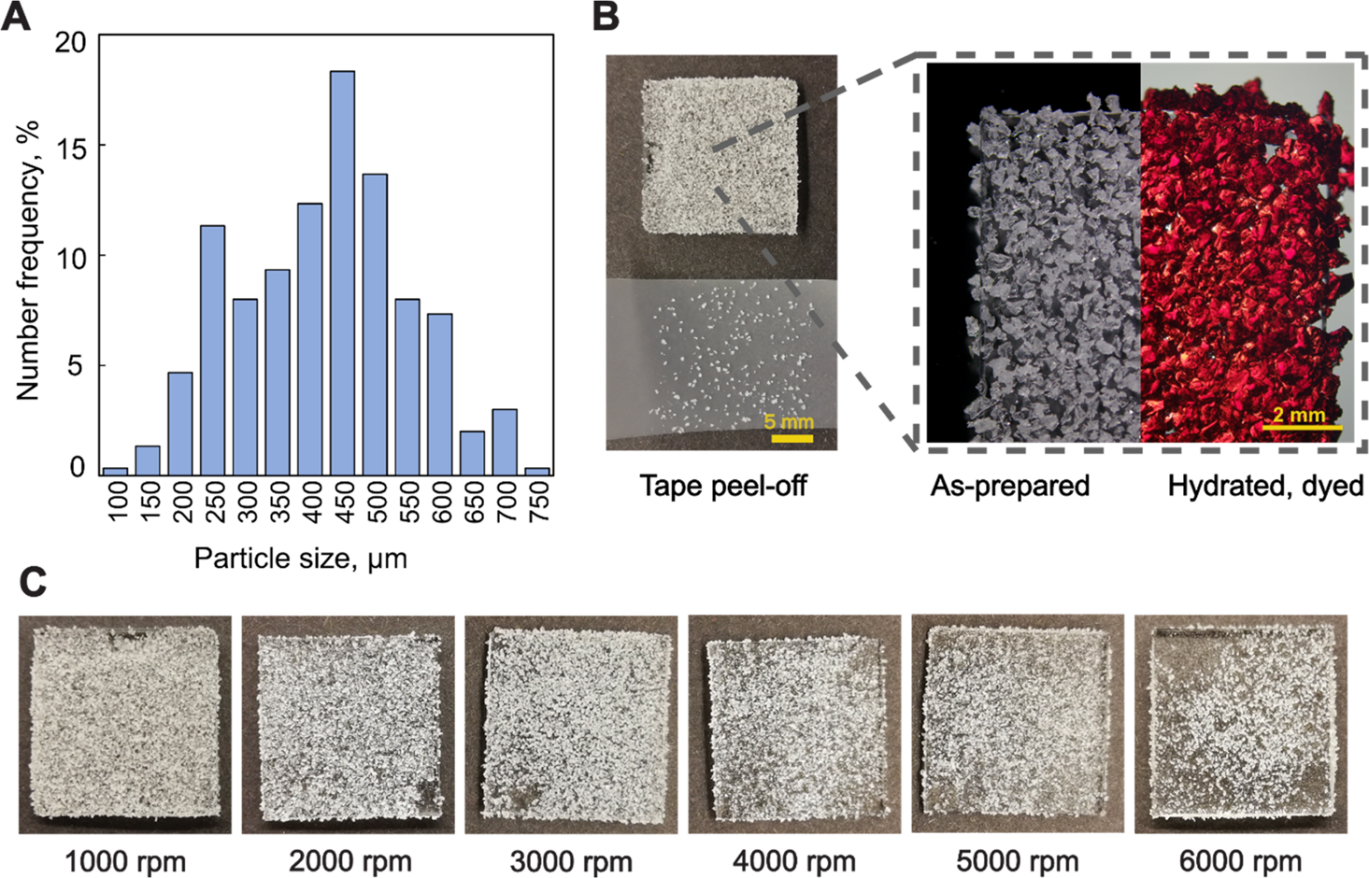
(A) As-prepared particle coating size distribution. (B, left) Photograph of the as-prepared particle coating at 3000 rpm and Scotch tape peel-off test example. (B, right) Stereomicroscopy image of the as-prepared coating and rehydrated coating with red safranine dye for increased contrast. (C) Spin coating speed effect on the coating stability evaluated by the Scotch tape peel-off test on as-prepared coatings, photographs of coatings after four consecutive tape applications and removals.

To estimate the microparticle size constituting the prepared coatings, particle size distribution was measured from the stereomicroscopy images by randomly selecting 300 particles (Figure 3A). Deposited particle size was estimated to range from approximately 100 to 750 μm with the average particle size around 450 μm as determined from the histogram.

To evaluate the functional performance of the coating, the as-prepared coatings were rehydrated in aqueous media. It has been previously estimated that 30% w/w DA-F127 bulk hydrogels have a swelling capacity up to 90% of the initial dry weight in water.^25^Figure 3B demonstrates rehydration of the as-prepared coating in aqueous solution of red safranine dye. It was observed that after rehydration the as-prepared coatings demonstrated visible swelling of the hydrogel particles and the expansion of the polymer network with no significant detachment and coating delamination, resulting in near full PDMS substrate coverage.

To estimate coating adhesion in the dry state, a qualitative assessment of the freeze-dried particle coating was performed by peel-off evaluation. Due to difficulties defining the contact area of the coating, conventional peel-off tests that may assess monolith coating adhesion were deemed unapplicable in this microparticle coating evaluation.^40^ Therefore, the Scotch tape peel-off test was used to assess the particle adhesion strength depending on the PDMS spin rate (Figure 3C).^41^Figure 3C shows the effect of four consecutive tape applications and removals on the stability of the coating. At spin speed up to 3000 rpm, as-prepared coatings exhibited satisfactory stability without significant reduction in the coating coverage. Further increase in the spin rate followed subsequent increase in particle detachment. At spin rates above 5000 rpm, large sections of the coating were detached by the tape, indicating weak physical adhesion and insufficient network entanglement. The results from the SEM and the peel-off tests point toward the reduction of IPN formation between the PDMS and hydrogel particles at high spin speeds and subsequently reduced coating stability.

As to coatings stability in rehydrated state, at spin rates between 1000 and 3000 rpm, the particle coating remained stable after swelling and multiple soaking steps like the ones used in microbiology and drug delivery tests, demonstrating the IPN effect on coating stability.

Ultimately, differences in coating adhesion indicated that PDMS spin rate could be used as a reliable parameter for controlling the mechanical stability of the particle coatings. However, further investigation is required to investigate the relation between additional downstream properties such as antimicrobial effect and its relation to the coating speed.

#### 3.2.1 Physiochemical Characterization of AMP Particle Coating

Coating characterization studies were performed to demonstrate the AMP incorporation in the particle coating using UV–vis spectroscopy, WCA analysis, Raman spectroscopy, and XPS.

Prior to the AMP particle coating preparation and following the AMP functionalization of the particles, the AMP content in the particle structure was quantified using UV–vis spectroscopy, with estimated 3.3% w/w AMP present in the particle structure with respect to dry polymer weight.

**Figure 4:**
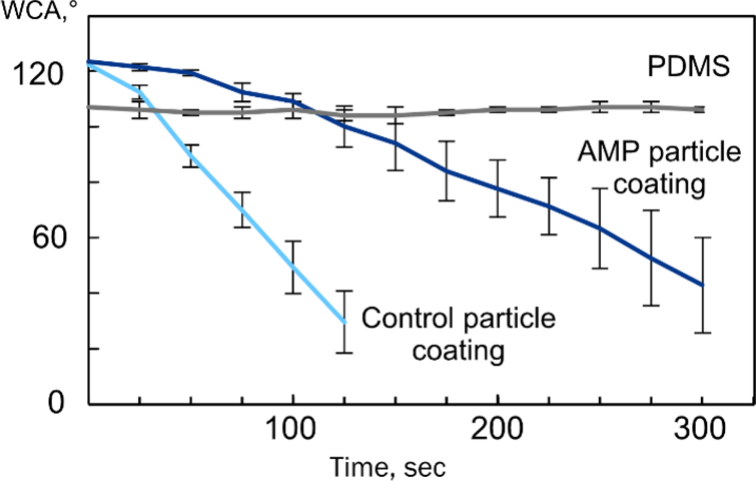
WCA analysis on as-prepared control particle coating, AMP particle coating, and pristine PDMS surface. Contact angle reduction demonstrates the particle swelling from water uptake. A slower swelling rate in AMP particle coating compared to control particle coating indicative of the AMP presence, *n* = 6.

Water contact angle analysis was performed to assess coating wetting properties of the as-prepared particle coatings with and without AMP and compared to the pristine PDMS surface (Figure 4). Pristine PDMS exhibited hydrophobic character consistent with the reported data with stable WCA of 105.7 ± 0.9° over the measurement period. The as-prepared particle coatings displayed increased initial hydrophobicity compared to PDMS of 122.3 ± 2.2° and 123.4 ± 3.1° for AMP-free control particle coating and AMP particle coating, respectively, most likely stemming from the amphiphilicity and surface roughness of the coatings. Both coating groups exhibited similar surface wetting profiles over time with decreasing contact angle, consistent with the swelling of the DA-F127 hydrogel network and water uptake in the hydrophilic domains of the LLC particle structure. The presence of AMP in the particle structure resulted in pronounced increase in wetting time, with approximately three-times longer for the AMP particle coating to achieve similar WCA compared to the control particle coating (from 30.6 ± 11.3° at 125 s for control particle coating to 43.3 ± 16.5° at 300 s for AMP particle coating, respectively). This observation may be associated with the presence of the hydrophobic tryptophan amino acid end-tag in the AMP structure, indicating particle functionalization with AMP.

**Figure 5:**
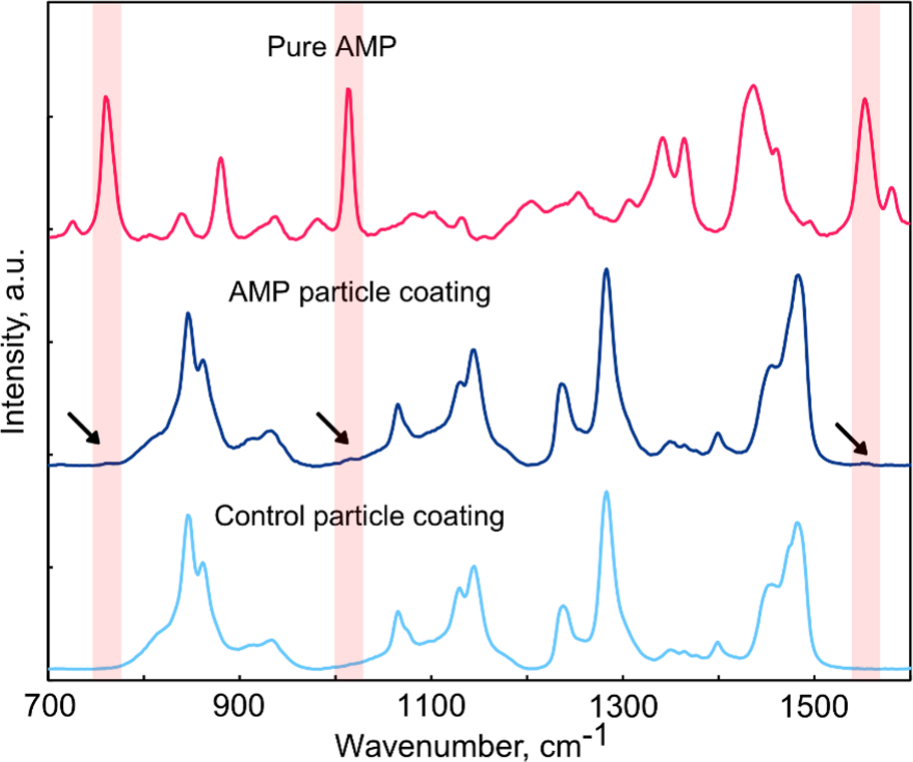
Raman spectra of control hydrogel particle coating, AMP particle coating, and pure AMP powder for reference. Characteristic absorption signals of AMP are marked with black arrows.

To further confirm peptide integration in the AMP particle coatings, Raman spectroscopy analysis was carried out on the as-prepared samples comparing the control hydrogel particle coating to AMP particle coating using pure AMP powder as a reference (Figure 5). Three small bands at 760 cm^–1^, 1015 cm^–1^, and 1552 cm^–1^ were observed in the Raman spectrum of the AMP particle coating that coincided with strong characteristic absorption bands present in pure AMP while absent in control particle coating. Absorption bands at 760 and 1015 cm^–1^ can be ascribed to benzene and pyrrole ring breathing vibrations in the bicyclic indole ring structure present in the tryptophan end tag of the AMP structure.^42,43^ Similarly, the 1552 cm^–1^ band can be ascribed to the stretching vibration of benzene and pyrrole rings in the tryptophan indole group, indicating successful AMP integration in the AMP particle coating.

XPS analysis was carried out to further identify the elemental surface composition of hydrogel particle coating, AMP particle coating, and pristine PDMS surface. Survey scans and high-resolution scans in the N 1s region were recorded (Figure S1), and the quantitative surface composition was determined (Table S1). Both control hydrogel particle coating and AMP particle coating showed increase in carbon (C 1s) concentration compared to the pristine PDMS, confirming the surface modification with the DA-F127-based particle coating. The presence of N 1s signal in the high-resolution spectra of the AMP particle coating could be observed while absent in the control particle coating (see Figure S1B,C). The corresponding nitrogen concentration was determined to be 0.2 atomic percent, further confirming AMP presence on the AMP particle coating surface. However, the low AMP quantity in the particle bulk (∼3% w/w), along with possible AMP reorientation in dry samples, must be considered as a contributing factor for the relatively weak signal in both Raman and XPS.

The principal mechanism of EDC/NHS activation protocol for AMP attachment utilized in our study relies on formation of direct peptide bond between the AMP and the particle surface and is therefore referred to as the “zero-length crosslinker”. Regarding the most likely intermediate products formed during the carboxyl group activation (*O*-acylisourea and NHS intermediate), they are released into the reaction medium upon AMP attachment and, in our case, removed by extensive washing of the particles before AMP attachment. The high water solubility of EDC, NHS, and the EDC isourea byproduct generated during the reaction further contributes to any excess reagent removal. Although it should be recognized that XPS cannot definitively demonstrate the AMP presence solely based on the N 1s signal, the Raman results give a more specific molecular signal of AMP. By viewing these results together with the antibacterial effect presented in the following section, a clear indication of AMP presence onto the particle coating can be made.

### 3.3 Antibacterial Activity of the AMP Particle Coating

**Figure 6:**
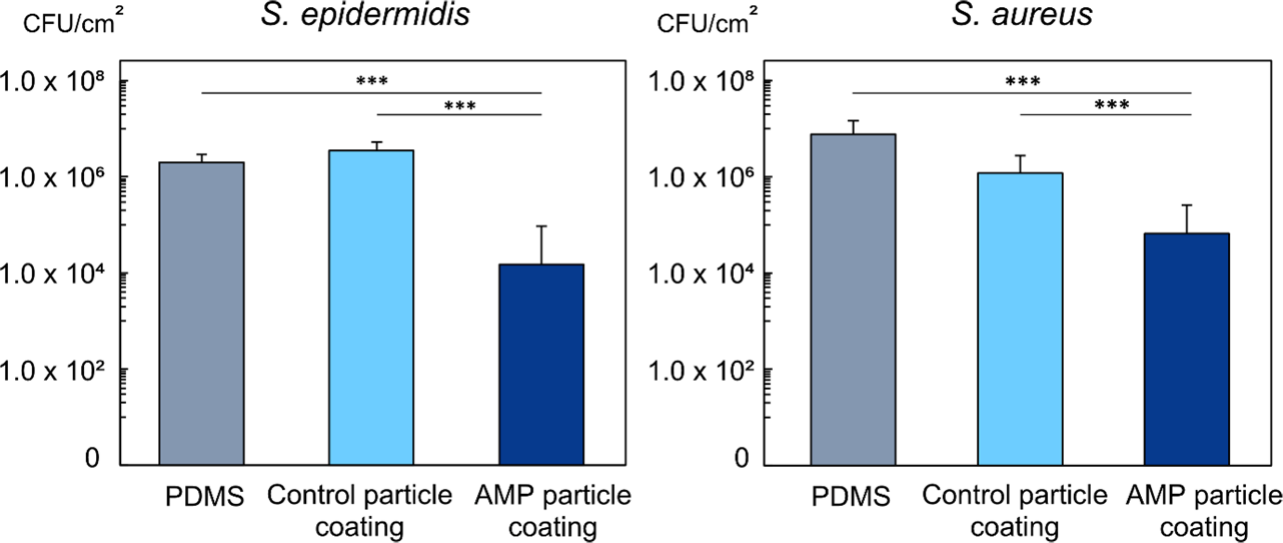
AMP particle coating antibacterial activity against *S. epidermidis* and *S. aureus*. Data expressed as CFU/cm^2^ based on the projected coating area, *n* = 9.

The antibacterial activity of the manufactured AMP particle coating was evaluated against two common Gram-positive pathogens: *S. epidermidis* and *S. aureus*. Pristine PDMS surface and control hydrogel coatings were used as negative controls in order to assess the AMP particle coating capacity to reduced bacterial attachment and proliferation on the coating surface. The samples were incubated overnight in the presence of *S. epidermidis* or *S. aureus* according to methods stated elsewhere, followed by the quantification of surface adhered live bacterial cells via colony forming unit counting.^44^ AMP particle coatings demonstrated significant reduction in the surface-adhered bacteria counts when compared to pristine PDMS and AMP-free control particle coating (Figure 6). Covalent attachment of AMP onto the hydrogel particle coating had a marked effect on the antibacterial activity against *S. epidermidis*, with reduction in live bacteria count by about 99.6% (2.4 log, *p* < 0.001) compared to control coatings and 99.3% (2.1 log, *p* < 0.001) in comparison to pristine PDMS. Similarly, high antibacterial activity was observed against *S. aureus* with AMP particle coating exhibiting reduction in bacterial viability by 94.5% (1.3 log, *p* < 0.001) compared to control coatings and 99.1% (2.1 log, *p* < 0.001) in comparison to pristine PDMS.

The killing mechanism of membrane active AMPs like RRP9W4N is driven by the electrostatic and hydrophobic interactions between the AMP and the bacterial membrane.^24^ Membrane active AMPs are known to exhibit strong electrostatic attraction to the negatively charged bacterial membrane, followed by the hydrophobic peptide domain penetration into the phospholipid bilayer, facilitating membrane disruption and cell death.^45,46^ Previous studies utilizing DA-F127 hydrogels and hydrogel particles with covalently immobilized RRP9W4N have clearly demonstrated the contact killing effect exerted by AMP by using live/dead imaging^25^ along with MIC and cryogenic transmission electron microscopy.^26^ Additionally, similar studies with contact killing AMP surface modifications have relied on CFU as the antibacterial activity evaluation method.^47,48^ Considering this, it can be argued that strong contact killing antibacterial effect can be achieved from the AMP particle coating as seen from the CFU results.

**Figure 7:**
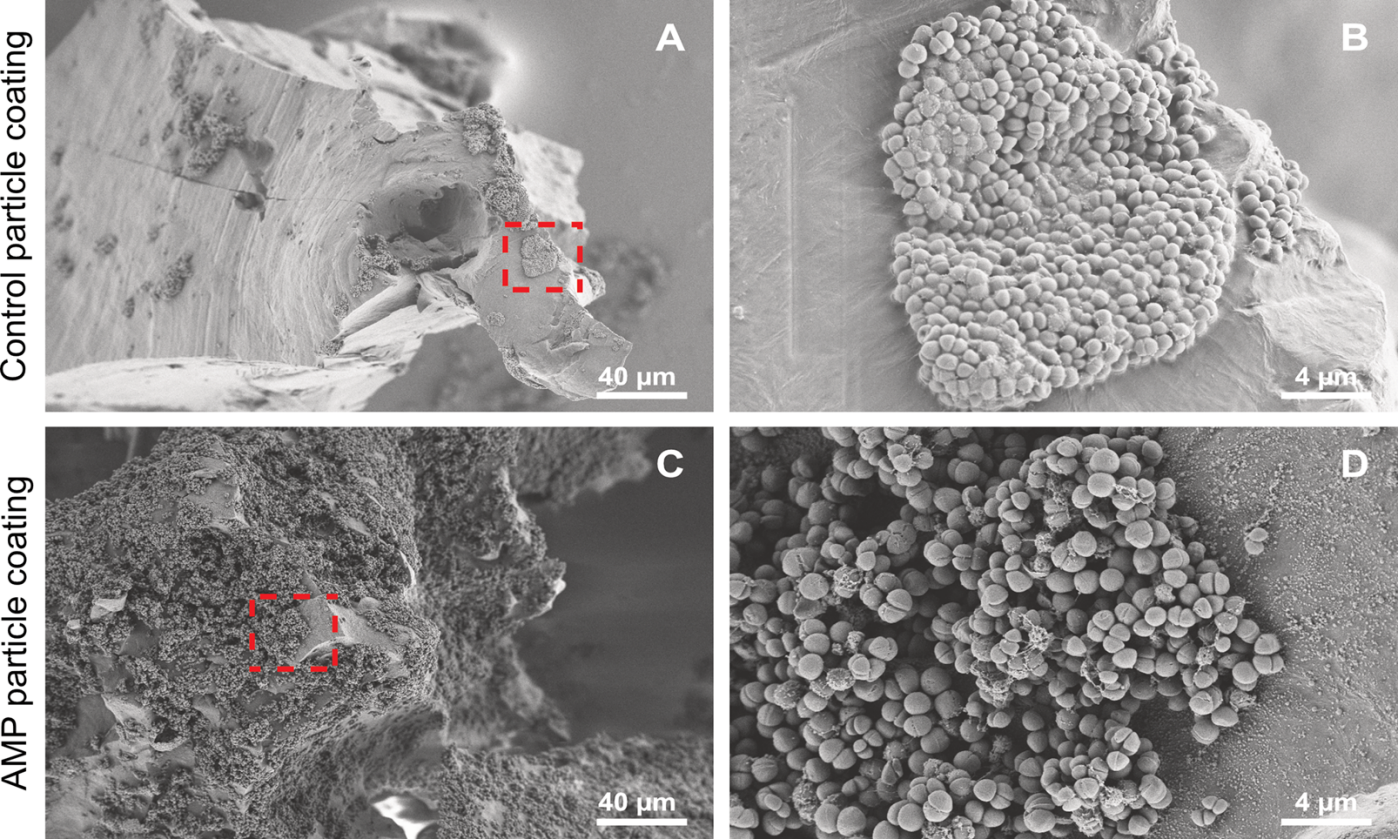
SEM micrographs of coating samples after overnight incubation with *S. epidermidis*. (A, B) Control particle coating and (C, D) AMP particle coating. AMP effect on bacteria attraction, attachment, and proliferation visible.

To further demonstrate the effect covalent AMP attachment has on the bacterial attachment and proliferation, particle coating samples were challenged with high bacterial concentration (10^8^ CFU/mL) of *S. epidermidis* and incubated overnight, followed by bacteria fixation and SEM imaging (Figure 7). Notable differences could be observed between the bacterial growth behavior on AMP-free control particle coating (Figure 7A,B) and AMP particle coating (Figure 7C,D). Bacteria exhibited a cluster-like growth behavior consistent with early stage biofilm formation on AMP-free control particle coatings. Conversely, AMP particle coating surface showed high bacterial saturation with significantly higher bacterial coverage, lacking the characteristic cluster-like growth behavior.

The drastic increase in the number of bacteria present on the AMP particle surface can serve as a direct indicator to the electrostatic attraction of the AMP, hence enforcing the contact killing activity hypothesis. However, it should be recognized that the SEM images cannot give any direct indication of the AMP activity and cannot be compared directly to the CFU results. Due to the bacterial concentration differences utilized in both studies, SEM can only serve as a qualitative indicator of the AMP electrostatic attraction effect.

Additionally, it should be noted that potential inhibition of the AMP by the underlying bacterial layer could lead to bacterial growth as evident by the binary fission seen in the AMP particle sample. It is to be expected that upon increase in bioburden (CFU/mL) AMP could lose its effect if bacteria have deposited onto an already dead cell layer. This is inevitably the theoretical limitation of contact killing surfaces and should be recognized. Although an interesting research question, experimental investigations of AMP attachment concentration versus bioburden, and the resulting antibacterial effect, are out of the scope of this study.

Altogether, the antibacterial activity evaluation of the AMP hydrogel microparticle coatings on PDMS suggests high antibacterial potential against *S. epidermidis* and *S. aureus*, mirroring previous findings on AMP contact-killing effect, both when covalently attached to bulk DA-F127 hydrogels^25^ as well as in hydrogel particle formulations.^26^ This study demonstrates an early proof-of-concept about the potential of AMP particle coating to endow the PDMS surface with antibacterial properties.

### 3.4 Drug Delivery from the Particle Coating

**Figure 8:**
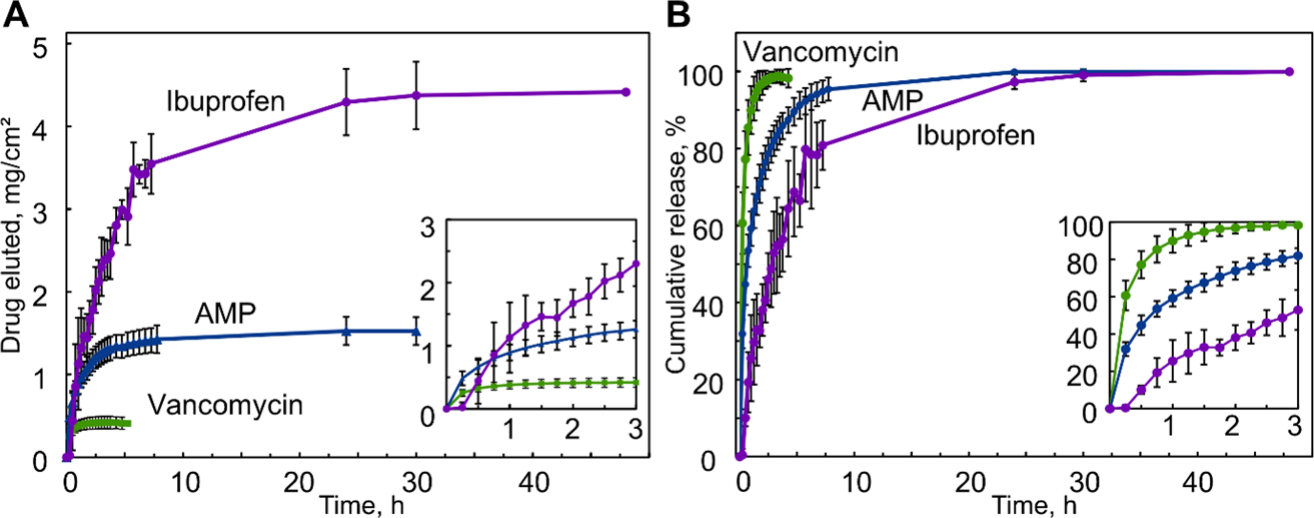
Drug release profiles of VCM and AMP in MQ water, and IBP in 1% w/v SDS buffer from the hydrogel particle coatings prepared onto PDMS substrates. (A) Results expressed as milligrams of drug eluted per cm^2^ of projected coating area, and (B) estimated cumulative release in % assuming equilibrium concentration of 100%. Insets demonstrate the first 3 h of measurement period, *n* = 9.

To demonstrate the hydrogel particle coating ability to encapsulate and release substances of different polarity, three different model drugs were chosen, i.e., VCM, AMP, and IBP as polar, amphiphilic, and nonpolar examples, respectively. VCM is a potent glycoprotein antibiotic used in treatment of Gram-positive bacterial infections with high water solubility (50 mg/mL), whereas IBP belongs to a class of nonsteroidal anti-inflammatory drugs with poor water solubility (0.011 mg/mL).^30^ The AMP solubility in water may be considered as average at ≤10 mg/mL, as reported by the manufacturer. In the present coating system, it was hypothesized for the hydrogel microparticles to be able to selectively entrap the drugs in the different polarity domains of the micellar cubic LLC structure, acting as drug depots for further delivery. The different *in vitro* release profiles generated are presented in Figure 8 with respect to absolute release in milligrams normalized to projected coating area and the estimated cumulative release in percentage. Notable differences in drug release behavior can be seen depending on the drug polarity.

As evident in Figure 8, VCM produced a predominant burst release profile with approximately 60% drug released in the first 15 min, following a rapid saturation around 2 h and reaching an equilibrium concentration of 0.410 ± 0.073 mg/cm^2^ or 0.082 ± 0.015 mg/mL. Although VCM demonstrated a low uptake into the coating samples, the eluted concentration at equilibrium exceeded minimum inhibitory concentration (MIC) for VCM-sensitive *S. aureus* (MIC ≤ 0.002 mg/mL) 33.5–48.5-times within the standard deviation, indicating a sufficient loading with regards to therapeutic requirements.^49^

In the case of AMP-loaded hydrogel particle coatings, a relatively sustained release profile could be observed. While still displaying a rapid burst release in the first measurement point with approximately 30% of drug released in first 15 min, slower release rate could be observed with equilibrium concentration of 1.526 ± 0.168 mg/cm^2^ or 0.313 ± 0.040 mg/mL reached in around 8 h. Similar to VCM, coatings exhibited therapeutically relevant AMP release dose, with concentration at equilibrium exceeding MIC 22.8–29.4-times (MIC for *S. aureus* ≈ 0.012 mg/mL). It is noteworthy that, although subjected to the same drug concentration in the loading solution feed, AMP uptake and release were significantly higher compared to those of the VCM. A probable reason for this includes the hydrophobic interactions between the tryptophan group residues in the AMP structure and the PPO segments in the DA-F127 structure contributing to prolonged AMP retention.

A different drug release profile was recorded for IBP, with the absence of an initial burst, but a sustained release pattern for up to 30 h. By using acetone as the solvent for the loading solution and increasing the feed concentration (1% w/v for VCM and AMP, 7% w/v for IBP), a subsequent increase in the maximum amount of eluted drug can be seen with 4.373 ± 0.402 mg/cm^2^ or 0.331 ± 0.031 mg/mL IBP eluted after 30 h. It is worth noting that a significant IBP release was detected in the control experiment with plain PDMS substrate; results absent in the case of VCM and AMP. The coating-free PDMS substrates alone were capable of delivering 0.604 ± 0.072 mg/cm^2^ of IBP after 30 h, indicating the hydrophobic drug uptake and retention in the PDMS matrix with sustained elution pattern (Figure S2). Regardless, the introduction of the hydrogel particle coating significantly increased the delivered IBP dose, demonstrating improved material functionality. To facilitate the IBP solubility and remove the dissolution as the limiting factor in the drug elution, 1% w/v aqueous SDS solution was utilized as the elution buffer to increase IBP solubility with micellization as the main driving force. Previously reported data yield IBP solubility to approximately 2.3 mg/mL in highly concentrated SDS solutions, hereby confirming that the investigated system operates under the sink condition.^50,51^

The stark difference in the IBP release profile compared to VCM and AMP could be attributed to (a) differences in the loading solution concentration, (b) acetone as the loading solvent leading to increased swelling of the constructs and potentially increased drug load in the material structure, and (c) SDS significantly facilitating IBP solubility. SDS impact on IBP solubility has been studied previously, with formation of mixed SDS and IBP micelles identified as the contributing factor in solubility enhancement. In a comprehensive study by K. Stoyanova et al., it was found that at a constant SDS conc. of 0.5% w/w (here SDS 1% w/v), IBP solubility was increased by a factor of 200, compared to an aqueous phase, potentially contributing to the IBP release differences found in our study.^31^

#### 3.4.1 Drug Release Kinetics

With experimental results clearly demonstrating effect of drug chemical polarity on the release behavior, experimental data were mathematically fitted to commonly utilized mathematical models to characterize drug release kinetics and elucidate the release mechanisms. Zero-order, first-order, Korsmeyer-Peppas, and Higuchi models were applied in their simplified mathematical expressions (Table 1).^52^

**Table 1:** Kinetic and Fit Data to the Mathematical Models for the Experimental VCM, AMP, and IBP Release

		Vancomycin				AMP				Ibuprofen			
kinetic model	equation^a^	*K*	*n*	*R*^2^	RMSE	*K*	*n*	*R*^2^	RMSE	*K*	*n*	*R*^2^	RMSE
zero-order	*Q*_*t*_ = *Q*_0_ + *K*_0_*t*	15.660		0.737	6.273	6.635		0.757	8.398	3.856		0.538	16.798
first-order	*Q*_*t*_ = *Q*_0_*e*^–*K*_1_*t*^	1.777		0.987	1.246	0.447		0.977	2.582	0.241		0.988	2.723
Korsmeyer-Peppas	*Q*_t_/*Q*_∞_ = *K*_P_*t^n^*					58.980	0.42	0.991	1.174	22.802	0.73	0.967	3.094
Higuchi		53.690		0.810	5.690	33.800		0.831	7.006	27.140		0.828	10.265

a Where *Q*_*t*_ is amount of drug released at time *t*, *Q*_0_ is the initial amount of drug release, *Q*_∞_ is the amount of drug released at equilibrium, *K*_0_ is the zero-order release constant, *K*_1_ is the first-order release constant, *K*_P_ is the Korsmeyer-Peppas constant incorporating structural and geometrical release parameter contribution, *K*_H_ is the Higuchi release constant, and *n* is a release exponent indicative of the drug release mechanism.

In the case of VCM, the release profile from the particle coatings best fitted the first-order release kinetics with *R*^2^ = 0.992 and lowest RMSE = 1.246 (see Table 1), indicating concentration-controlled release behavior. In fact, first-order release is expected in case of highly water-soluble drugs like VCM incorporated in porous hydrogel matrices under maximal solubility limit, resulting in rapid release.^53^ Zero-order and Higuchi models provided unsatisfactory fit, while the Korsmeyer-Peppas model could not be applied due to VCM reaching 60% elution in the first data point ( can be fitted to the Korsmeyer-Peppas model). Nevertheless, it is probable to assume the initial rapid release of VCM to be the hydrophilic DA-F127 segment swelling and relaxation controlled, with rapid solvent penetration and the hydrophilic PEO chain reorganization, followed by diffusion-controlled release through the aqueous channels. Compared to non-cross-linked Pluronic F127-VCM delivery systems reported in the literature, where polymer erosion is thought to a contributing factor in VCM release, no significant increase in VCM retention could be observed in the cross-linked DA-F127 version investigated in the current study.^54,29^

AMP release data displayed the best mathematical adaptation to Korsmeyer-Peppas model with *R*^2^ = 0.991 and RMSE of 1.174 as well as high correlation to first-order model. Korsmeyer-Peppas model is a semiempirical model established to describe drug release from porous hydrophilic polymers; however, it has been applied to a variety of modified release pharmaceutical dosage forms and is often used when the drug delivery mechanism in unknown.^55,56^ Here the diffusional exponent *n* can be used to assess the delivery mechanism. For *n* = 0.50, Fickian diffusion via solvent penetration is the governing force for solute diffusion, while at *n* = 1.0 (Case II transport), the drug delivery is irrespective of solvent diffusion but limited by polymer relaxation and swelling rate, also known as zero-order time-independent release. For 0.50 < *n* < 1.0, non-Fickian or anomalous transport takes place through simultaneous diffusion and polymer chain relaxation in a time-dependent manner. According to model calculations, *n* for AMP release equals 0.42, indicative of quasi-Fickian release behavior via diffusion. Although initially developed for drug release from thin film delivery systems, the Korsmeyer-Peppas model has been further adapted for other geometries, e.g., cylindrical and spherical systems with estimated *n* values of 0.45 and 0.43, respectively, indicating Fickian diffusion.^57^ Considering the high heterogeneity of the hydrogel particle morphology, the estimated *n* value for AMP release thereby demonstrates good correlation to the form factor.

IBP demonstrated the best fit to the first-order release kinetics with *R*^2^ = 0.988 and RMSE = 2.723 similarly to VCM. In addition, the kinetic constant values showed good correlation to the experimental data with *K*_*1 IBP*_ = 0.241 exhibiting the lowest *K*_*1*_ compared to equivalent *K*_1 VCM_ = 1.777 and *K*_*1 AMP*_ = 0.447, indicating slowest drug release rate. The release exponent *n* was determined to be 0.73, descriptive of anomalous transport mechanism of diffusion and swelling combination. Considering the hydrophobic nature of IBP and its affinity toward the hydrophobic core of the LLC micelles, the *n* value points toward slow swelling of the hydrophobic segments in SDS and diffusion through the polymer matrix as the simultaneous driving factors for IBP elution.

**Figure 9:**
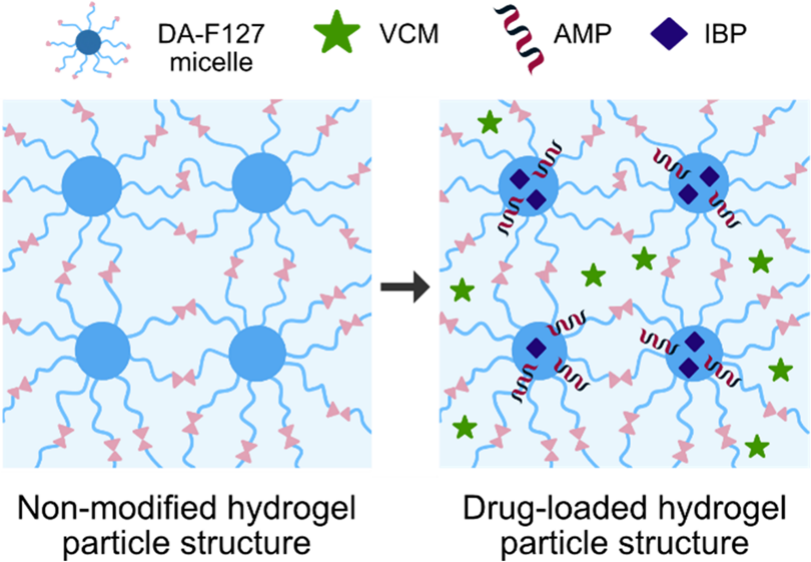
Schematic representation of the proposed location of VCM, AMP, and IBP in the micellar cubic DA-F127 hydrogel structure.

Ultimately, it was hypothesized that different drugs have selective preference toward the specific domains of the hydrogel microparticle structure with a proposed scheme seen in Figure 9. With VCM being the most polar of the drugs, its location is limited to the hydrophilic exterior of the micellar structure contrary to IBP being preferably taken up by the hydrophobic micellar interior. As discussed previously, the amphiphilicity of the AMP molecules serves for improved retention at the hydrophilic–hydrophobic micellar interface. The drug release experiments demonstrate the potential for the hydrogel microparticle coating to selectively encapsulate and release therapeutic drugs of different polarity from PDMS structures without additional modification, thus endowing the material with new functionalities.

## 4 Conclusions

This study describes the development of a PDMS surface modification strategy by fabrication of amphiphilic hydrogel-based microparticle coating. A physical immobilization method has been proposed via formation of interpenetrating polymer network between the PDMS and the amphiphilic hydrogel particles, resulting in stable coating formation without delamination. The coating functioned as a platform for covalent antimicrobial peptide (AMP) attachment exerting high antibacterial effect against *S. epidermidis* and *S. aureus*, common pathogens involved in medical device-associated infection. The physiochemical characterization via water contact angle analysis, Raman spectroscopy, and X-ray photoelectron spectroscopy confirmed PDMS surface modification with hydrophilic properties and covalent AMP attachment. Drug delivery studies demonstrated the hydrogel particle coating’s ability to encapsulate and release drugs of different polarity with vancomycin, AMP, and ibuprofen, as model polar, amphiphilic, and nonpolar drugs, respectively. An early proof-of-concept for facile strategy of PDMS surface modification with amphiphilic hydrogel particle coating is demonstrated, yielding dual material function of contact-killing antibacterial surface properties with a complementary drug delivery capacity.

## Supporting Information Available

XPS survey and high-resolution spectra, atomic concentration quantification on particle coating, AMP particle coating and pristine PDMS surfaces, drug release pattern of IBP from plain PDMS substrates (PDF)
