# Supplementary material for: Multifunctional Surface Modification of PDMS for Antibacterial Contact Killing and Drug-Delivery of Polar, Nonpolar, and Amphiphilic Drugs
Source: ACS Appl Bio Mater. 2022 Nov 2;5(11):5289–301. doi: 10.1021/acsabm.2c00705 (PMC9682518; doi:10.1021/acsabm.2c00705)
Supplement: Supplementary file 1 — mt2c00705_si_001.pdf [file mt2c00705_si_001.pdf]

Supporting Information

Multifunctional Surface Modification of PDMS for Antibacterial Contact Killing and Drug-Delivery of Polar, Nonpolar and Amphiphilic Drugs

Annija Stepulane[1,2], Anand Kumar Rajasekharan[2] and Martin Andersson* [1,2]

[1] Department of Chemistry and Chemical Engineering, Chalmers University of Technology, Gothenburg, SE-412 96, Sweden

[2] Amferia AB, AZ BioVentureHub, Mölndal, SE-431 83, Sweden

*Corresponding author email: martin.andersson@chalmers.se

No. of pages: 2

No. of figures: 2

No. of tables: 1

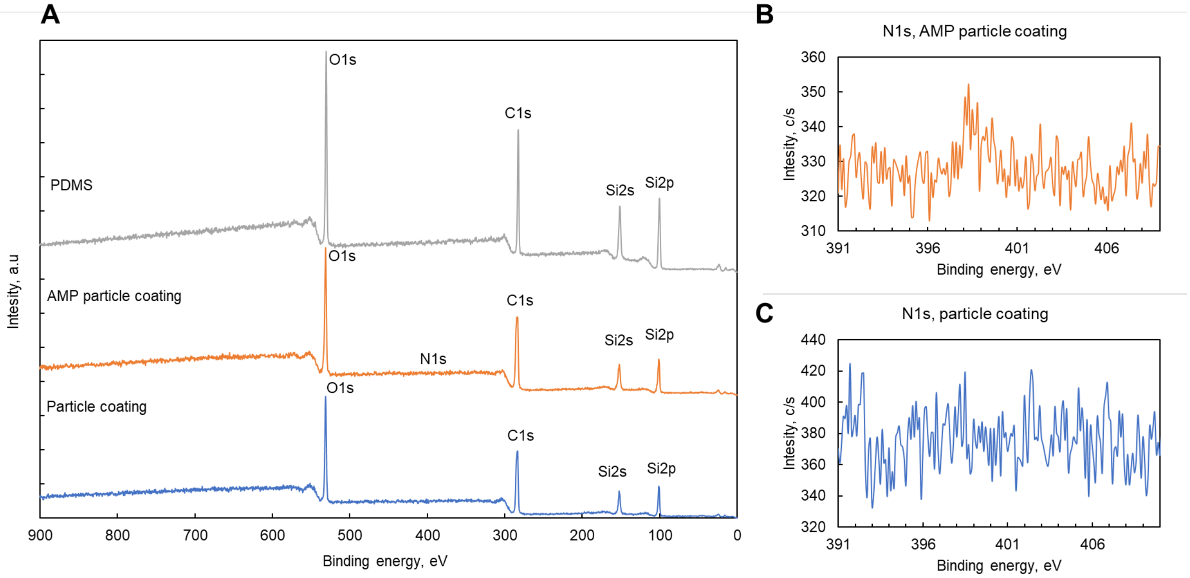

Figure S1. (A) XPS survey scans of particle coating, AMP particle coating and pristine PDMS surfaces. (B) High resolution scan of N1s on AMP particle coating. (C) High resolution scan of N1s on particle coating. The presence of N1s on AMP particle coating indicates presence of AMP.

Table S1. Surface elemental composition in atomic percent determined by XPS

	**Atomic concentration (%)**			
	**C 1s**	**O 1s**	**Si 2p**	**N 1s**
PDMS	44.8	31.1	24.1	-
Particle coating	57.2	28.9	13.9	-
AMP particle coating	56.7	29.1	14.0	0.2

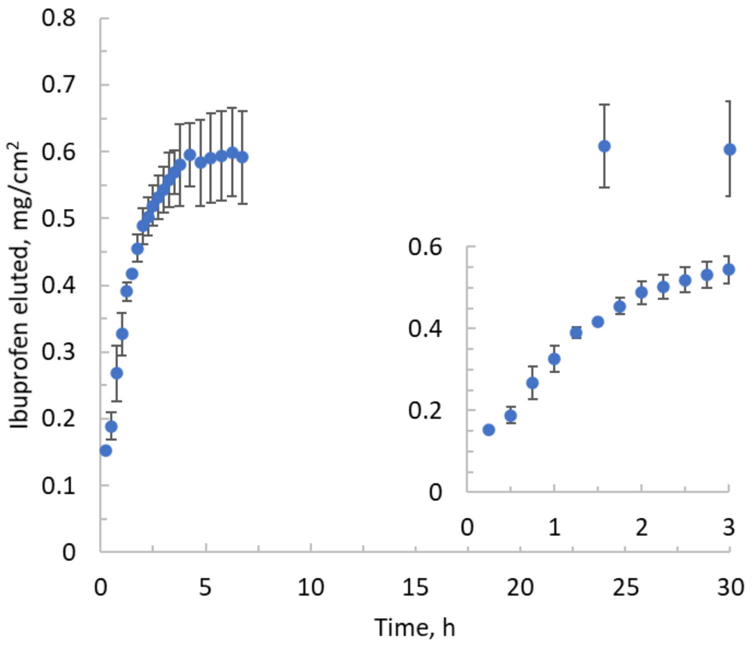

Figure S2. Drug release profile of IBP in 1% w/v SDS buffer from plain PDMS substrates. Results expressed as milligrams of drug eluted per cm^2 of projected sample area. Inset demonstrates the first 3 h of measurement period, n = 3.
